# Ferroptosis-related long non-coding RNA signature predicts the prognosis of hepatocellular carcinoma: A Review

**DOI:** 10.1097/MD.0000000000031747

**Published:** 2022-11-25

**Authors:** Fan Bu, Shijie Yin, Ruiqian Guan, Yao Xiao, ShuLin Zeng, Yonghou Zhao

**Affiliations:** a Heilongjiang University of Chinese Medicine, Haerbin, China; b The Second Affiliated Hospital of Heilongjiang University of Chinese Medicine, Haerbin, China.

**Keywords:** HCC, ferroptosis-related, lncRNA, prognosis

## Abstract

Hepatocellular carcinoma (HCC) is a liver cancer. In contrast, ferroptosis is a novel iron-dependent and ROS reliant type of cell death that is observed under various disease conditions.

**Methods and analysis:**

RNA sequencing data from HCC patients were downloaded from The Cancer Genome Atlas (TCGA) database. Ferroptosis-related long non-coding RNAs (lncRNAs) were screened by Pearson correlation analysis. Patients were randomized into training or testing sets in a 1:1 ratio. They were constructed in the training set using univariate-Lasso and multivariate Cox regression analysis and further tested for prognostic values in the testing set. Four lncRNAs were identified. Kaplan–Meier analysis showed that patients in the high-risk group had a worse prognosis than those in the low-risk group. Following differentially expressed genes analysis of these two groups. Functional analysis showed association with oxidative stress response. Cox regression analyses showed that risk score was an independent prognostic indicator. Receiver operating characteristic curve (ROC) and decision curve analysis demonstrated the accuracy of prediction. Four ferroptosis-related lncRNAs based on differential expression of HCC were screened by bioinformatic methods to construct a prognostic risk model and accurately predict the prognosis of HCC patients. Four lncRNAs may have a potential role in the anti-tumor immune process and serve as therapeutic targets for HCC. To lay the foundation for subsequent studies.

## 1. Introduction

Hepatocellular carcinoma (HCC) is one of the most common malignancies in the world, ranking in the top 10 in global mortality^[1]^ and the second highest patient mortality rate among the five most common cancers^[2]^ and has a recurrence rate of up to 70% after surgery.^[3]^ Ferroptosis is a cell death pathway that depends on intracellular reactive oxygen species and intracellular iron, unlike apoptosis and cellular autophagy, and is closely associated with cancer development and progression^[4]^ lncRNA is involved in several physiological processes in the body, and recent studies have shown that ferroptosis-related lncRNAs are associated with the prognosis of many cancers.^[5]^ The role of ferroptosis-related lncRNAs in HCC is currently unclear. In this study, we used bioinformatics to obtain ferroptosis-related lncRNAs that affect the prognosis of HCC, providing new ideas to further explore HCC and ferroptosis and to assess the prognosis of HCC.

## 2. Materials and methods

### 2.1. Data acquisition

RNA sequencing data of 424 was collected from the TCGA-HCC database. After removing incomplete data, Clinical data of TCGA-HCC were shown in Table 1. HCC-associated mRNAs and lncRNAs were identified. 259 ferroptosis genes were screened by downloading the ferroptosis database, including ferroptosis genes, ferroptosis-inhibiting genes and ferroptosis signatures, and removing duplicates and unleveled genes. They were provided in Supplemental Digital Content (Table S1, http://links.lww.com/MD/H905). We screened for 246 ferroptosis-related mRNAs in HCC provided in Supplemental Digital Content (Table S2, http://links.lww.com/MD/H906). Then, after Pearson correlation analysis, the correlation between the amount of ferroptosis-related genes expression and lncRNAs were calculated, with the conditions of *P *< .001 and absolute value of correlation coefficient > 0.6, and 127 ferroptosis-related lncRNAs were screened in HCC described in Supplemental Digital Content (Table S3, http://links.lww.com/MD/H907). The clinical and pathological data of HCC patients were collated, including gender, age, tumor grade, clinical stage, TMN stage, survival status and survival time.

**Table 1:** Clinical data of TCGA-HCC (n = 337).

Variable	Categories	Number of cases
Sex	M/F	230/107
Age	≤65/>65	218/119
Grade	G1/G2/G3/G4	45/166/114/12
Stage	I/II/III/IV	168/81/83/4
T	T1/T2/T3/T4	170/83/74/10
M	M0/M1/NA	258/3/76
N	N0/N1/NA	247/4/86

HCC = hepatocellular carcinoma, TCGA = The Cancer Genome Atlas.

### 2.2. Construction of a prognostic risk model for ferroptosis-related lncRNAs

**Figure 1.**
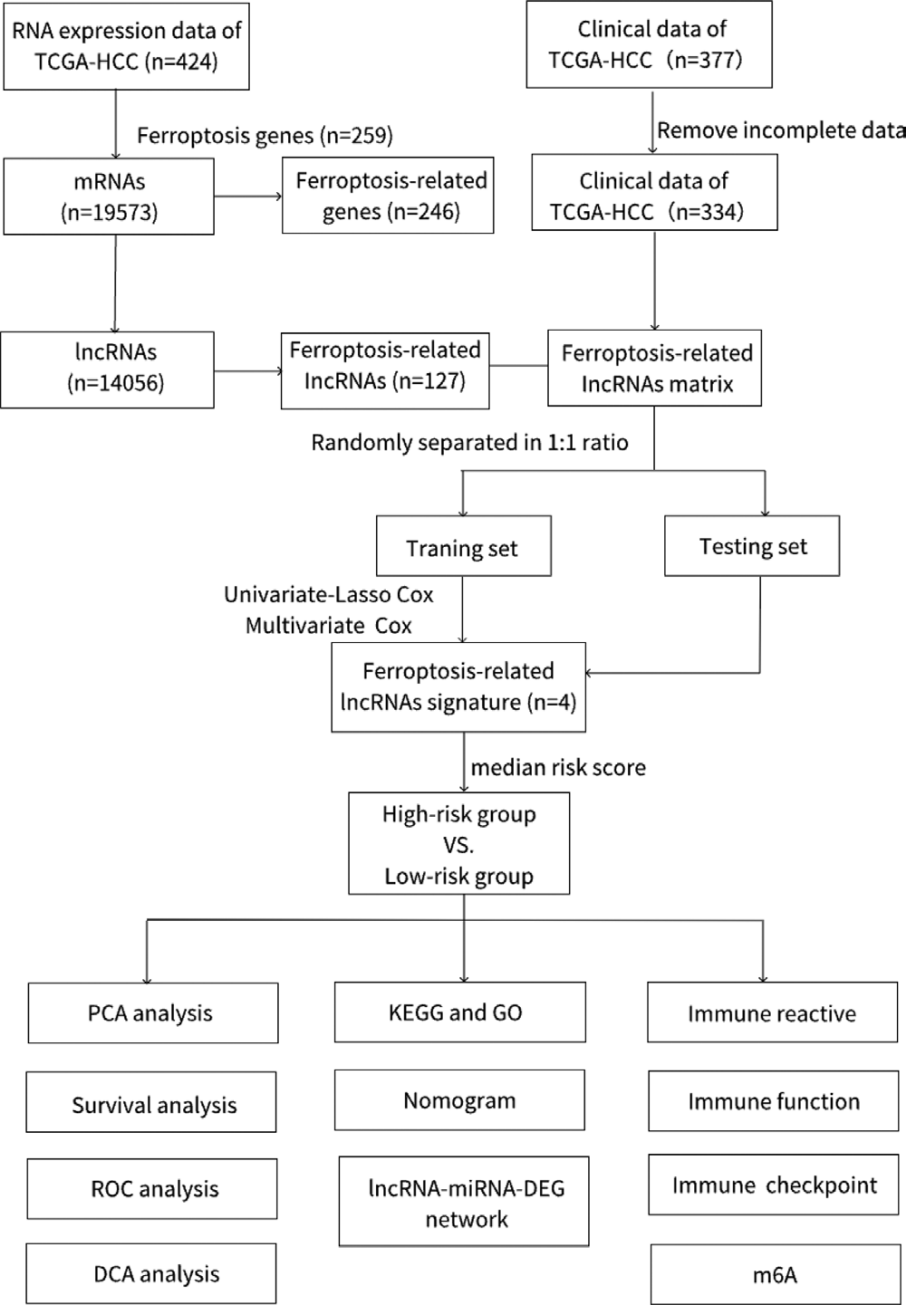
Flowchart. DCA = decision curve analysis, lncRNA = long non-coding RNA, GO = gene ontology, HCC = hepatocellular carcinoma, KEGG = Kyoto Encyclopedia of Genes and Genomes, ROC = receiver-operating characteristic curve, TCGA = The Cancer Genome Atlas.

The flow chart is shown in Figure 1. The 342 HCC patients were randomly divided into training and testing sets in a 1:1 ratio, and the prognostic value of each lncRNA in the training set was assessed by univariate Cox regression analysis (*P* < .05), with further screening based on least absolute shrinkage and selection operator (Lasso) regression to avoid overfitting. Next, to determine the final prognostic ferroptosis-related lncRNAs, multivariate Cox regression analysis was used.

Riskscore = βlncRNA1 × ExpressionlncRNA1 + βlncRNA2 × ExpressionlncRNA2 + βlncRNA3 × ExpressionlncRNA3 + ... + βlncRNAn × ExpressionlncRNAn.

### 2.3. Prediction capacity of risk score model and statistical analysis

Patients in the training and testing sets were divided into high- and low-risk groups using median risk score values and the corresponding coefficients for the training set. Survival curves were generated using the Kaplan–Meyer method. Predictive power was assessed by evaluating the receiver operating characteristic (ROC) curve and the area under the ROC curve (area under curve). ROC and decision curve analysis (DCA) were used to assess the sensitivity and specificity of prognostic features derived from HCC compared to other clinicopathological features.^[6]^ The testing set and the entire set were applied to validate this model. Univariate-Lasso Cox regression and multivariate Cox regression analyses were performed to assess the prognostic value of the risk score model. *P* < .05 was set as statistically significant, and FDR-q < 0.25 was used as a screening condition to construct nomograms in combination with prognostic features for predicting survival of HCC patients at 1-, 3-, and 5-years.

### 2.4. Functional analysis and lncRNA-miRNA-DEG co-expression network

To investigate differences between high- and low-risk groups at gene expression, we performed differentially expressed genes (DEGs) analysis with the criteria of |log2 (fold change) | (|(FC)|)> 1 and *P* < .05. The Gene Ontology (GO) and Kyoto Encyclopedia of Genes and Genomes (KEGG) were performed by R packages. Associated miRNAs were predicted with 4 lncRNAs and DEGs by DIANA Tools,^[7]^ miRcode,^[8]^ miRbase^[9]^, ENCORI and miRwalk.^[10]^ Cytoscape software was applied to visualize the lncRNA-miRNA-DEG co-expression network to be used to predict the function of lncRNA and its upstream and downstream regulatory relationships.

### 2.5. Immunoassay and gene expression

Compare CIBERSORT,^[11,12]^ ESTIMATE,^[13]^ McCounter,^[14]^ and TIMER^[15]^ algorithms to assess the cellular component or cellular immune response between high- and low-risk groups based on the characteristics of ferroptosis-related lncRNAs. Heat maps were used to reveal the differences in immune responses under different algorithms.

## 3. Result

### 3.1. Prognostic analysis of ferroptosis-related lncRNAs

**Figure 2.**
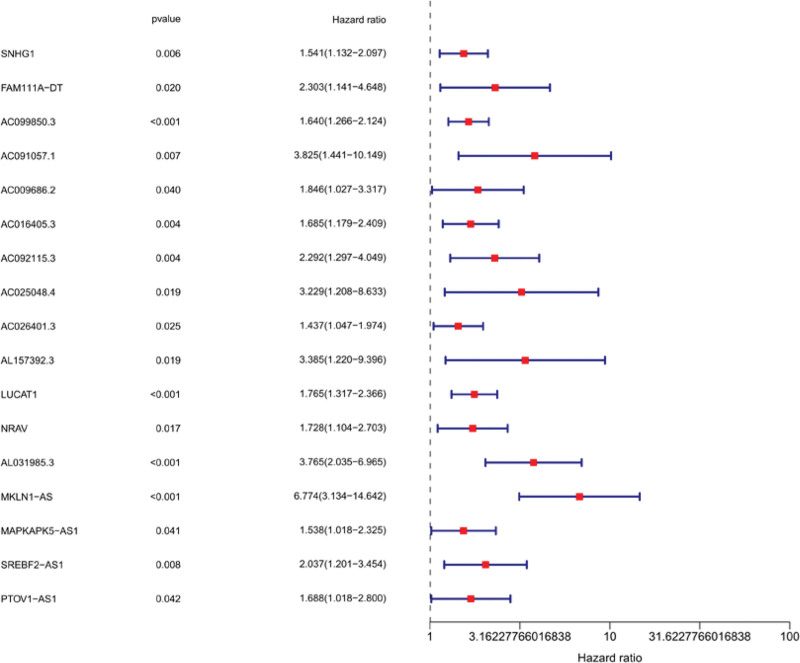
Univariate COX regression.

**Figure 3.**
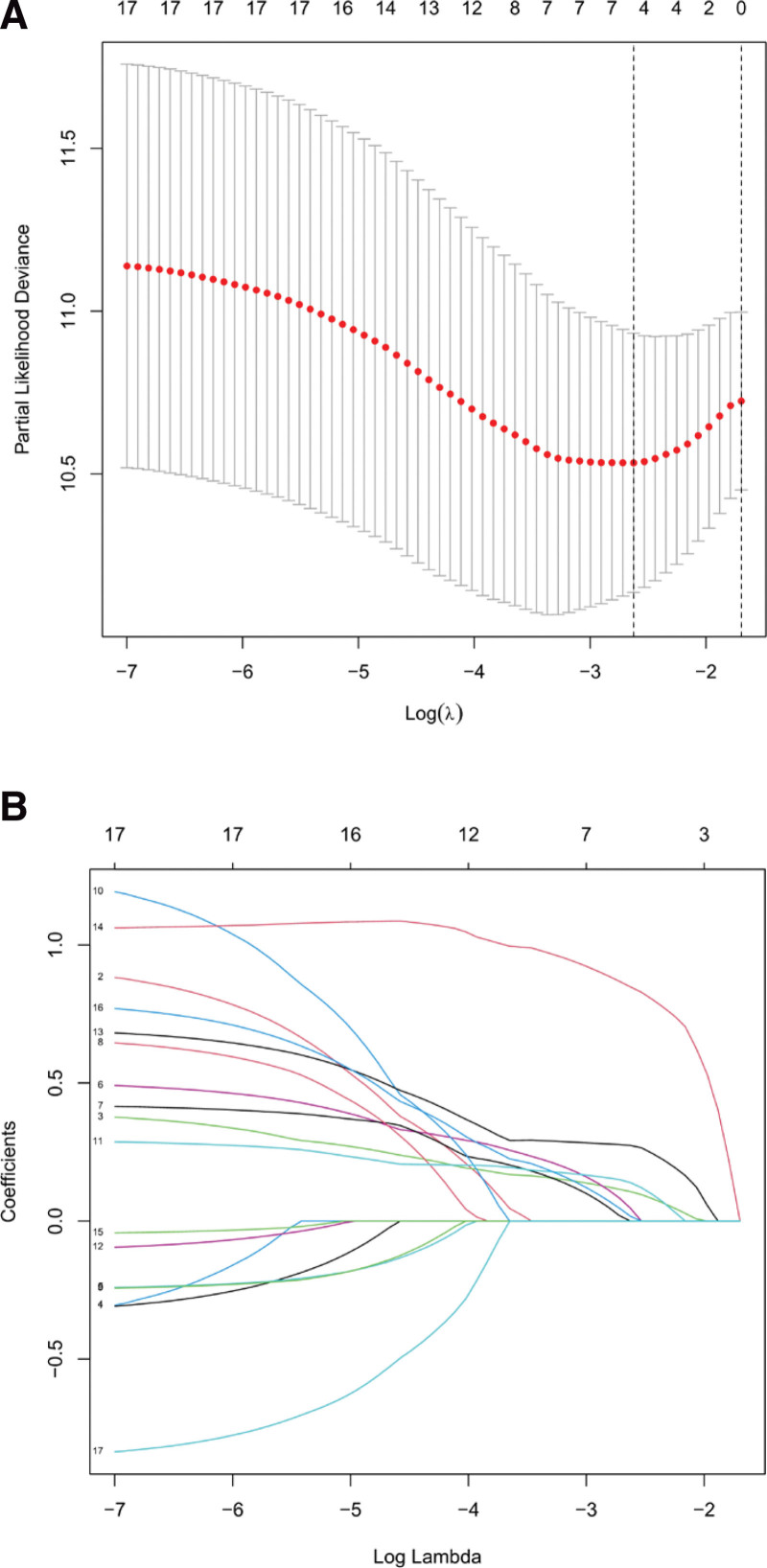
Lasso regression.

**Figure 4.**
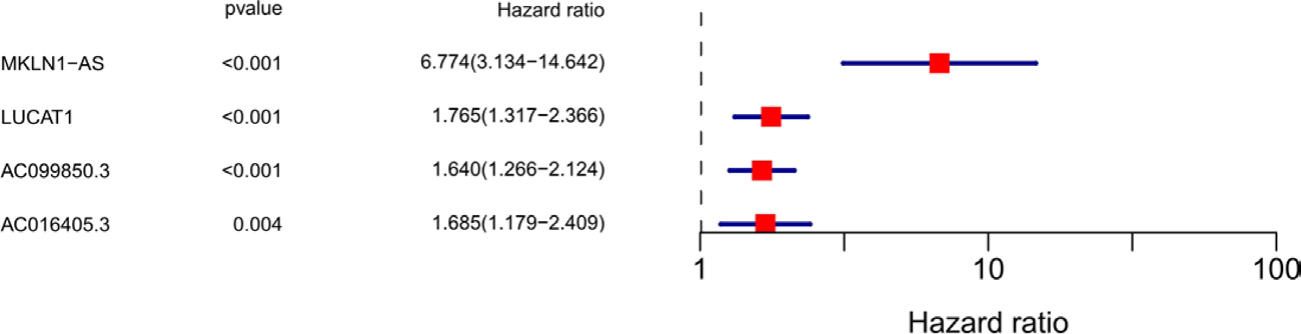
Multiivariate COX regression.

Seventeen ferroptosis-related lncRNAs were obtained by preliminary univariate Cox analysis (Fig. 2). The results were then further screened by Lasso regression analysis to identify 4 lncRNAs (Fig. 3). The final multivariate Cox regression analysis showed that these 4 ferroptosis-related lncRNAs could be independent prognostic factors for HCC patients (Fig. 4).

### 3.2. Survival analysis and multivariate analysis

**Figure 5.**
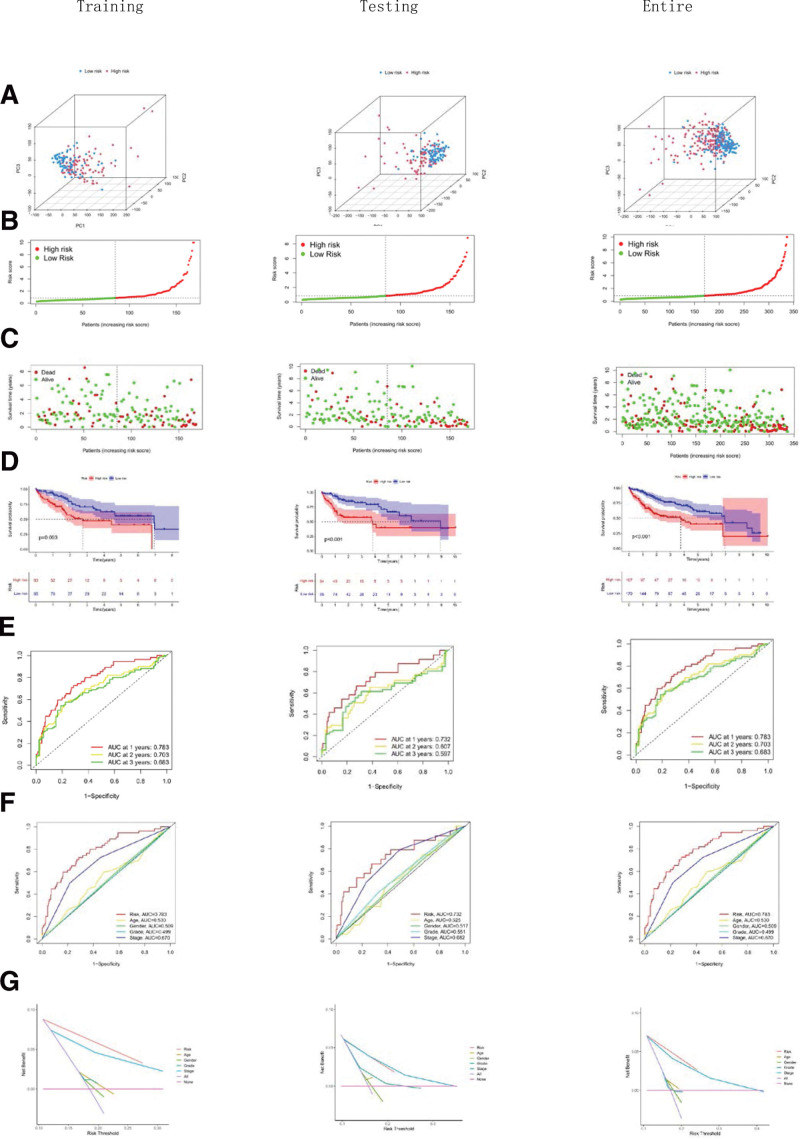
Validation of the prognostic risk model in HCC. (A) PCA shows the distinguished distribution of high- and low-risk patients based on the risk model. (B) Risk score curve of the high- and low-risk patients in the training, testing, and entire sets. (C) Patients‘ status in the training, testing, and entire sets. (D) The overall survival of the high- and low-risk patients in the indicated training, testing, and entire sets. (E) Time-dependent ROC curves (1, 2, 3 years) analysis for survival prediction verified the prognostic performance of the risk-score model in the indicated training, testing, and entire sets. (F) The ROC curves exhibit superior performance of risk-score compared to other measured characteristics in the indicated training, testing, and entire sets. (G) The DAC curves exhibit superior performance of risk-score compared to other measured characteristics in the indicated training, testing, and entire sets. DEG = differentially expressed gene, HCC = hepatocellular carcinoma, PCA = principal component analysis, ROC = receiver operating characteristic curve.

To assess the accuracy of stratification, we performed a principal component analysis (Fig. 5A), risk score analysis (Fig. 5B) and survival status analysis (Fig. 5C) first. The results showed that risk scores were effective in distinguishing high-risk patients from low-risk groups in TCGA training, testing, and entire set. Kaplan–Meier survival analyses showed that survival was significantly lower in the high-risk group than in the low-risk group in both the training, testing and entire (*P* < .003, Fig. 5D). Also, the ROC curves showed that the 1-, 2-, and 3-years survival area under curves were almost all greater than 0.6 (Fig. 5E). Compared with traditional clinicopathological features, ROC with DCA had more accurate results in predicting HCC prognosis (Fig. 5F and G). These results suggest that these 4 lncRNAs risk models have good predictive power and stratification accuracy for HCC patients.

### 3.3. Independent prognostic value of the 4-ferroptosis-related lncRNAs signature

**Figure 6.**
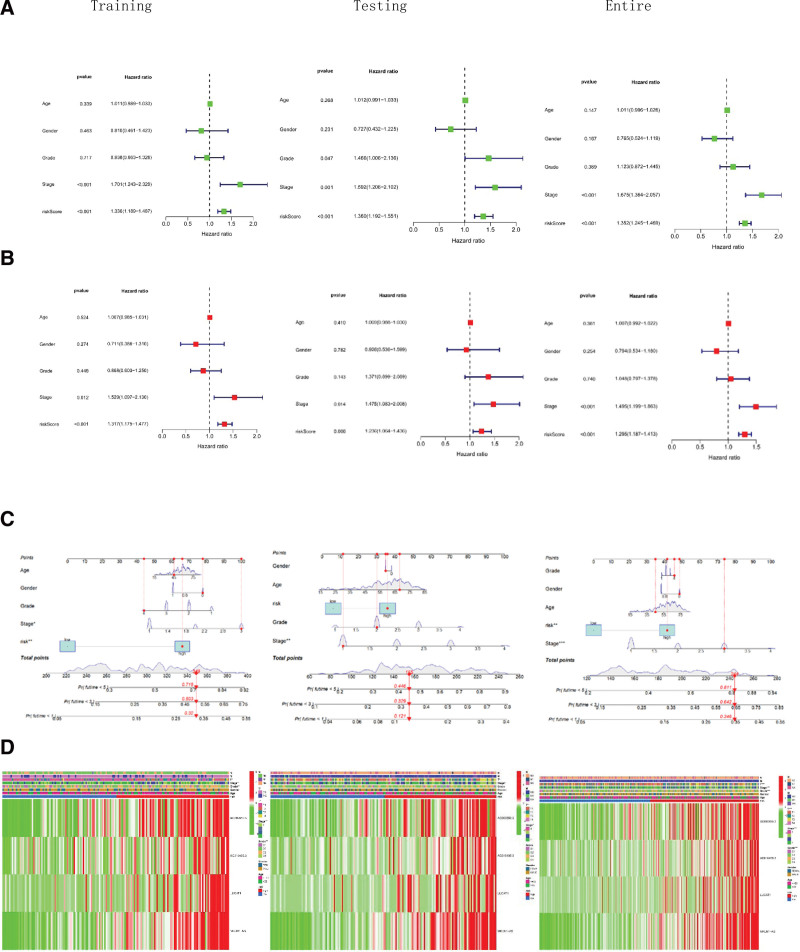
Independent prognostic value of the ferroptosis-related lncRNAs signature. (A) Univariate Cox regression analysis in the indicated training, testing, and entire sets. (B) Multivariate Cox regression analysis in the indicated training, testing, and entire sets. (C) Construction of a prognostic nomogram based on the risk score and clinicopathological parameters to predict 1-, 3-, and 5-years in the indicated training, testing, and entire sets. (D) Clinical heatmap of 4 ferroptosis-related lncRNAs in the indicated training, testing, and entire sets. lncRNA = long non-coding RNA.

To determine whether risk score is an independent prognostic factor for patients with HCC, univariate Cox regression analysis and multivariate Cox regression analysis were performed on clinical characteristics and risk score. The results showed that risk scores were highly associated with HCC in the training, testing and entire sets (Fig. 6A and B). Subsequently, clinicopathological characteristics (including age, gender, grade and stage and risk score) were applied to construct column line plots to predict the prognosis of HCC patients (Fig. 6C). In addition, heat maps correlating ferroptosis-related lncRNA prognostic features with clinicopathological manifestations were generated (Fig. 6D).

### 3.4. Functional analysis and lncRNA-miRNA-DEG co-expression network

**Figure 7.**
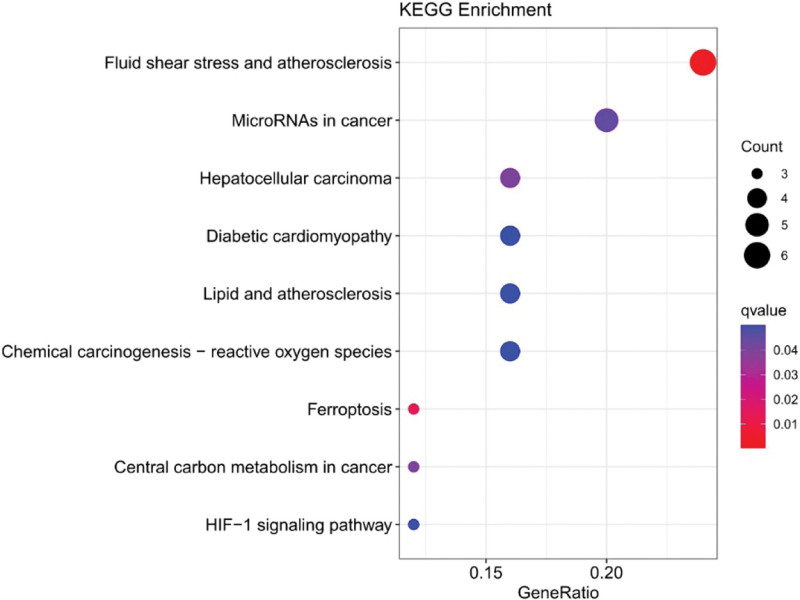
KEGG analyses for ferroptosis-related DEGs in HCC. DEG = differentially expressed gene, HCC = hepatocellular carcinoma, KEGG = Kyoto Encyclopedia of Genes and Genomes.

**Figure 8.**
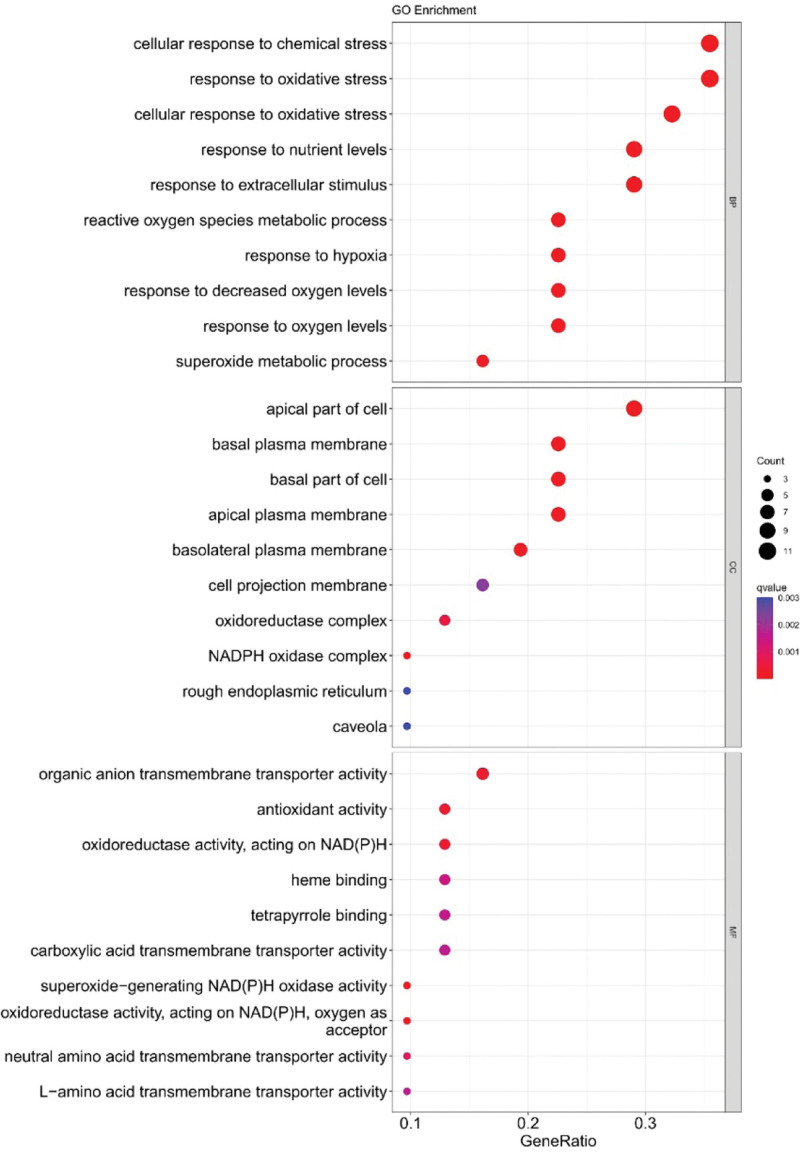
GO analyses for ferroptosis-related DEGs in HCC. DEG = differentially expressed gene, GO = gene ontology, HCC = hepatocellular carcinoma.

We found 31 DEGs (up-regulation: 30 and down-regulation: 1) (Supplemental Digital Content (Table S4, http://links.lww.com/MD/H908)). Enrichment analysis of DEGs was performed to map the top 30 pathways (Figs. 7 and 8). GO functional enrichment analysis yielded 290 entries, including 240 biological process entries, 24 cell composition (CC) entries, and 26 molecular function entries. KEGG pathway enrichment screening yielded 9 signaling pathways. The involved biological process mainly included cellular response to chemical stress, oxidative stress response, and response to oxidative stress; CC mainly included apical part of cell, basal plasma membrane, and basal part of cell; molecular function mainly included antioxidant activity, organic anion transmembrane transporter activity, and oxidoreductase activity. Related pathways involved are mainly cancer and cardiovascular disease, such as atherosclerosis, MicroRNAs in cancer, HCC, diabetic cardiomyopathy, chemical carcinogenesis, Central carbon metabolism in cancer. Among them, HCC is consistent with the research direction of this experiment, indicating that the results have good reliability and the relationship between oxidative stress response and HCC can be further considered.

**Figure 9.**
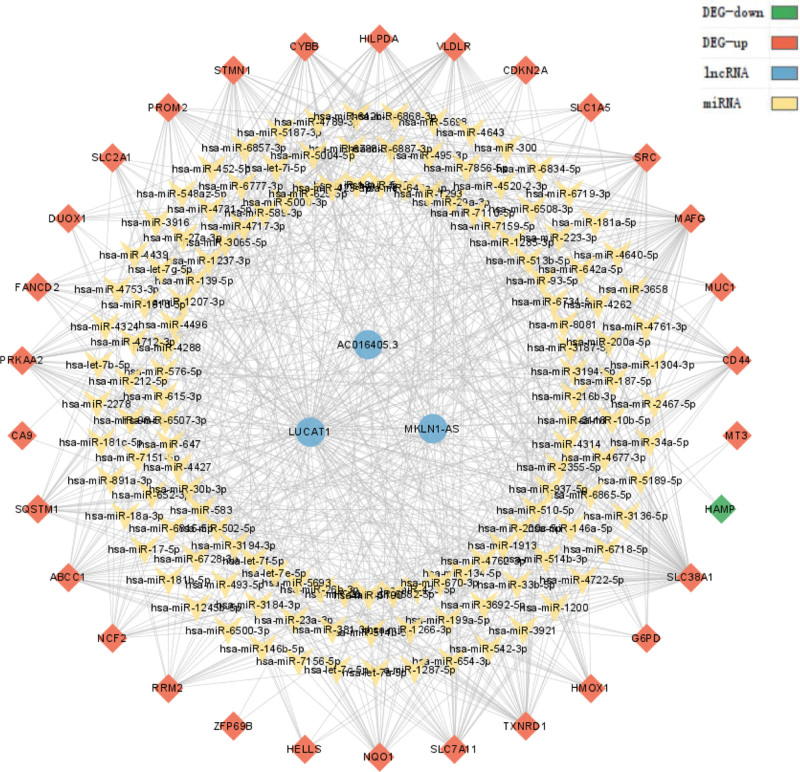
lncRNA-miRNA-DEG network. DEG = differentially expressed gene, LncRNA = long non-coding RNA.

We predicted relationship among lncRNAs, miRNAs, and DEGs by constructing co- expression networks to be used to predict the function of lncRNA and its upstream and downstream regulatory relationships (Fig. 9). Unfortunately, we looked through a large database and still did not find miRNAs corresponding to AC099850.3. However, it is undeniable that AC099850.3 is undeniably associated with proliferation, apoptosis, migration, and invasion proliferation of HCC and may be a potential biomarker and therapeutic target for HCC.^[16,17]^

### 3.5. Immunity and Gee expression

**Figure 10.**
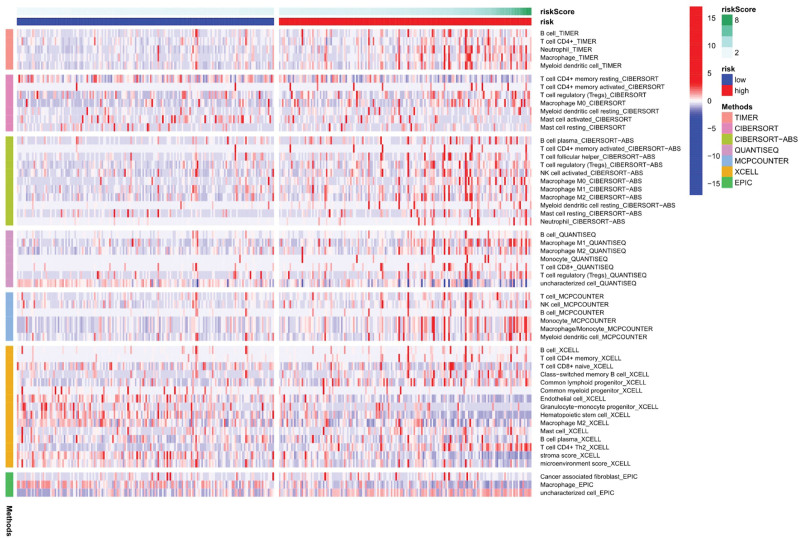
Immune reactive heat map.

**Figure 11.**
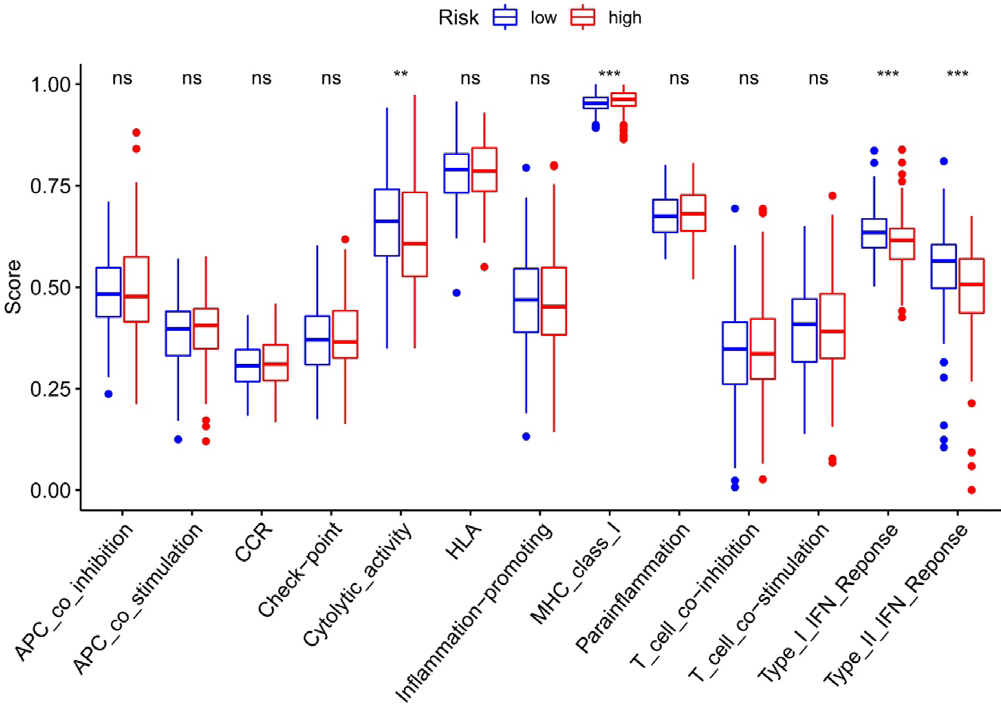
Immune function differential analysis.

**Figure 12.**
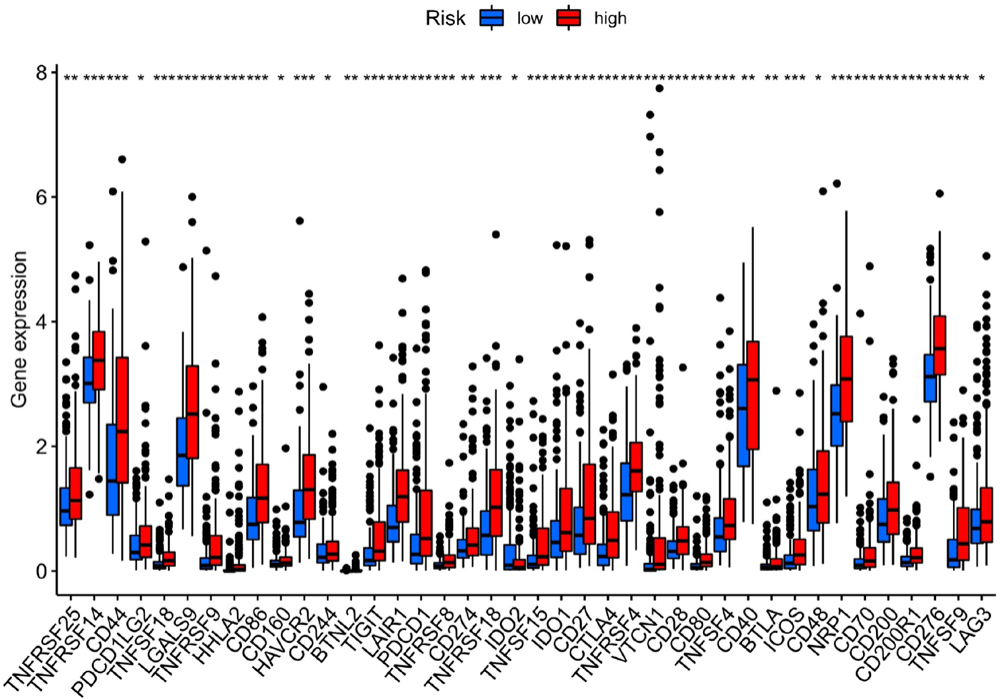
Immune checkpoint analysis.

**Figure 13.**
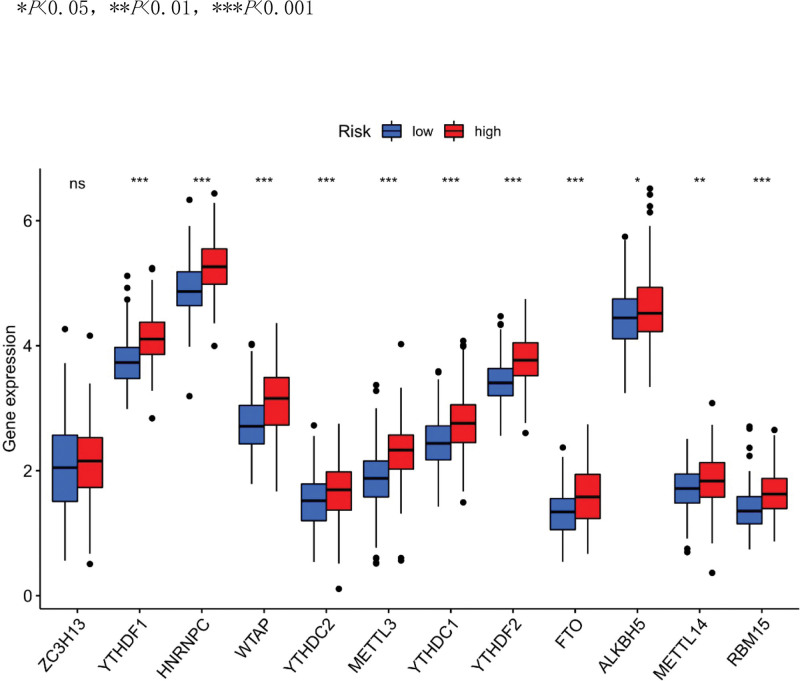
m6A analysis.

Immune response heatmaps were drawn based on four algorithms, CIBERSORT, ESTIMATE, MCP counter, and TIMER (Fig. 10). Correlation analysis showed significant differences in cytolytic activity, MHC class I molecules, type I and type II INF responses between the high-risk and low-risk groups (Fig. 11); immune checkpoints showed different levels of CD44, TNFRSF88 and CD27 (Fig. 12). Given the importance of checkpoint inhibitor-based immunotherapy, further analysis revealed significant differences in the levels of YTHDF1, METTL3, RMB15 (Fig. 13).

## 4. Discussion

We mainly used the method of validation in real in-house set, which has higher accuracy compared with direct prediction.^[18]^ Univariate-Lasso Cox regression can effectively avoid overfitting.^[19]^ The ROC model method is simple and intuitive, and the accuracy of the analysis learner can be observed and analyzed by graphical representation, and the judgment can be made by naked eyes. The ROC curvet line combines the true case rate and false positive case rate by a graphical method, which can accurately reflect the relationship between the true case rate and false positive case rate of a certain learner and is a comprehensive representative of the detection accuracy.^[20]^ The DCA model served as a supplement to further optimize the experimental structure.^[21]^ The above methods have been applied in related studies and have achieved good results.^[22–24]^

In our study, the role of immune infiltrating cells in the tumor microenvironment and immune checkpoint inhibitors in the prognosis of HCC was further explored by first characterizing ferroptosis-related lncRNAs based on differential expression of HCC based on the TCGA dataset. The findings of this study revealed potential biomarkers and therapeutic targets in the ferroptosis signaling pathway, and four differential expression of ferroptosis-related lncRNAs (AC099850.3, LUCAT1, AC016405.3, and MKLN1-A) were independent prognostic factors for HCC.

LUCAT1 is located in the nucleus and cytoplasm of cells and may play a regulatory function in many cancers, such as lung cancer,^[25]^ clear cell renal cell carcinoma^[26]^ colorectal cancer^[27]^ HCC.^[28]^ Jiao et al^[29]^ conducted a retrospective analysis of HCC patients and found that LUCAT1 was highly expressed in HCC tissues and its active expression was usually negatively correlated with the survival cycle of patients, so LUCAT1 was used as a biomarker to predict the prognosis of HCC patients. To further determine the mode of effect of LUCAT1 on HCC, Lou et al^[30]^ established a HCC xenograft model. It was shown that by upregulating the expressiveness of LUCAT1, the proliferation, migration and invasion degree of HCC cell lines could be significantly promoted, playing a key role in the development and metastasis of HCC. The transcription factor ETS proto-oncogene 1 plays an oncogenic role in different types of cancers, including HCC.^[31]^ Pan et al^[32]^ hypothesized that MKLN1-AS mediates the level of EST1 by binding miR-22-3p. MKLN1-AS levels were quantified and localized by collecting tissue samples from HCC patients using RT-qPCR. The dataset showed that MKLN1-AS was mainly located in the cytoplasm of HuH7 and LM3 cells, and the levels of MKLN1-AS were much higher in HCC tissues than in normal tissues. The constructed human tumor nude mouse xenograft model showed that silencing MKLN1-AS inhibited the proliferation, angiogenesis, migration and invasion of HuH7 and LM3 cells, confirming that MKLN1-AS functions as an oncogenic regulator in HCC. Guo et al^[33]^ analyzed the expression of MKLN1-AS and YAP1 in HCC patients based on several databases, and their tissues were cultured and examined to determine the location of MKLN1-AS in cells and the effect of MKLN1-AS on HCC patients. The results showed that the expression of MKLN1-AS was significantly higher in HCC tissues than in normal tissues and was mainly located in the cytoplasm of HCC cells. Overexpression of MKLN1-AS enhanced the stability of YAP1 mRNA and further accelerated the proliferation, migration and invasion of HCC cells. For normal tissues, MKLN1-AS also contributed to HCC development by inducing YAP1 expression in vivo. Therefore, MKLN1-AS can be used as an upstream factor of YAP1 for the diagnosis and prognosis of HCC.

## 5. Conclusions

Ferroptosis is a new form of cell death that may provide new avenues for tumor therapy. However, many critical issues, such as the interconnection of ferroptosis with other cell death and host immunogenicity, need to be urgently addressed. In this study, a novel model of ferroptosis lncRNAs was constructed to explore ferroptosis biomarkers that could help predict the prognosis of HCC, thus informing the therapeutic approach to the disease. In addition, based on limited clinical data, the prognostic prediction model developed in this study needs further validation.

## Author contributions

**Conceptualization:** Fan Bu, Shijie Yin.

**Funding acquisition:** Fan Bu.

**Methodology:** Ruiqian Guan.

**Software:** Yonghou Zhao.

**Visualization:** Shijie Yin, Shulin Zeng.

**Writing – original draft:** Fan Bu, Yao Xiao.

**Writing – review & editing:** Fan Bu.

## Supplementary Material
